# Phenotypic consequences of a nanophthalmos-associated *TMEM98* variant in human and mouse

**DOI:** 10.1038/s41598-023-37855-x

**Published:** 2023-07-07

**Authors:** Mark M. Hassall, Shari Javadiyan, Sonja Klebe, Mona S. Awadalla, Shiwani Sharma, Ayub Qassim, Melissa White, Paul Q. Thomas, Jamie E. Craig, Owen M. Siggs

**Affiliations:** 1 Department of Ophthalmology, Flinders University, Bedford Park, SA Australia; 2 Department of Anatomical Pathology, Flinders University, Bedford Park, SA Australia; 3 Department of Molecular and Cellular Biology and Robinson Research Institute, University of Adelaide, Adelaide, Australia; 4 Garvan Institute of Medical Research, Darlinghurst, NSW Australia

**Keywords:** Genetic engineering, Glaucoma

## Abstract

Nanophthalmos is characterised by shorter posterior and anterior segments of the eye, with a predisposition towards high hyperopia and primary angle-closure glaucoma. Variants in *TMEM98* have been associated with autosomal dominant nanophthalmos in multiple kindreds, but definitive evidence for causation has been limited. Here we used CRISPR/Cas9 mutagenesis to recreate the human nanophthalmos-associated *TMEM98* p.(Ala193Pro) variant in mice. The p.(Ala193Pro) variant was associated with ocular phenotypes in both mice and humans, with dominant inheritance in humans and recessive inheritance in mice. Unlike their human counterparts, p.(Ala193Pro) homozygous mutant mice had normal axial length, normal intraocular pressure, and structurally normal scleral collagen. However, in both homozygous mice and heterozygous humans, the p.(Ala193Pro) variant was associated with discrete white spots throughout the retinal fundus, with corresponding retinal folds on histology. This direct comparison of a *TMEM98* variant in mouse and human suggests that certain nanophthalmos-associated phenotypes are not only a consequence of a smaller eye, but that *TMEM98* may itself play a primary role in retinal and scleral structure and integrity.

## Introduction

Nanophthalmos is an inherited condition characterised by bilaterally small but structurally normal eyes, and associated with high hyperopia and an increased risk of angle-closure glaucoma. At least four genes have been associated with nanophthalmos, including recessive (*PRSS56*, *MFRP*) and dominant (*TMEM98*, *MYRF*) forms, with recessive forms phenotypically more severe than their dominant counterparts^1^.

Mouse models of human nanophthalmos are phenotypically diverse, and do not always mirror their human counterparts. A close murine counterpart to human nanophthalmos is the *Prss56*^*grm4/grm4*^ mutant, which exhibits reduced axial length, narrow drainage angles, ocular hypertension, and glaucomatous neurodegeneration^2,3^. Mouse *Mfrp* mutants, on the other hand, have a normal axial length, but are associated with white fundus flecks, progressive retinal degeneration, and retinal pigment epithelium atrophy^4,5^. Similarly, homozygous conditional deletion of *Myrf* in the developing retina was associated with patchy retinal degeneration, but no difference in ocular axial length^6^.

*TMEM98* was first associated with nanophthalmos in a large European kindred segregating the p.(Ala193Pro) missense variant^7^. Although the significance of this association had been questioned^8^, reports of *TMEM98* variants in multiple additional nanophthalmos kindreds have followed, including the p.(His196Pro) variant in a European family, and a 34 bp deletion spanning the 3’ end of exon 4 in a Micronesian family^9^. Subsequently, the same p.(Arg201Pro) variant has been reported in two apparently unrelated families in Austria^10^, and the US^11^. More common *TMEM98* variants have also been associated with myopia in genome-wide association studies^12,13^.

To further investigate the role of *TMEM98* in ocular development we used CRISPR/Cas9 editing to create a mouse model of human *TMEM98*-associated nanophthalmos. We report here the phenotypes observed in these mice, as compared to the effects of the corresponding variant in a human nanophthalmos case with the corresponding variant.

## Results

**Figure 1:**
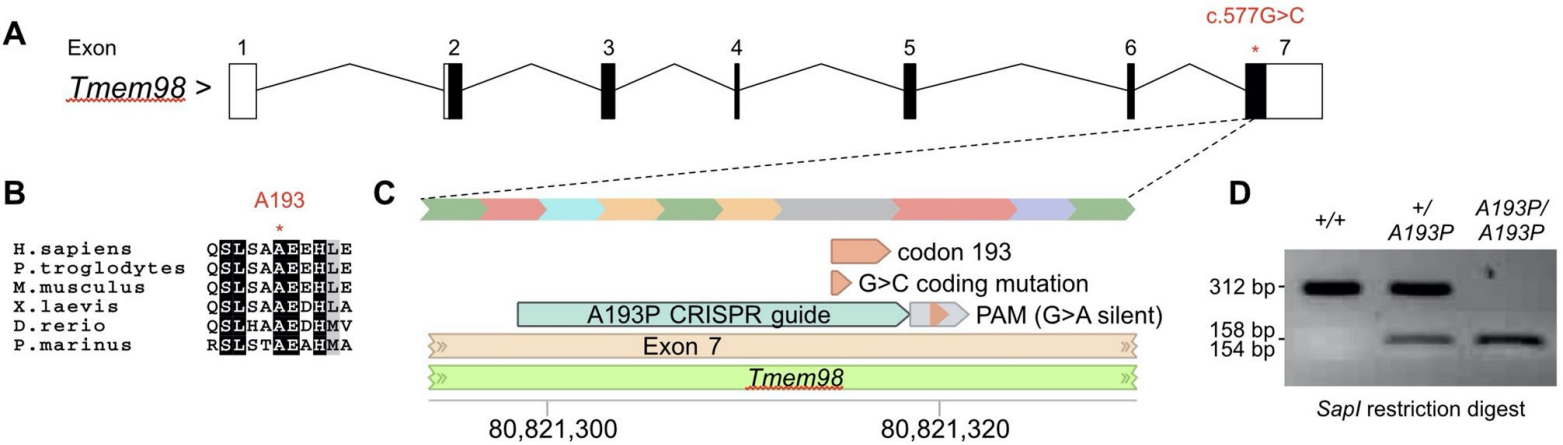
Creation of the *Tmem98*^*A193P*^ variant in mice by germline CRISPR/Cas9 editing. (**A**) Structure of the mouse *Tmem98* consensus transcript (ENSMUST00000040865.8) detailing coding (filled) and non-coding (unfilled) exons, and the location of the c.577G > C variant leading to the p.(Ala193Pro) (A193P) missense substitution. (**B**) Amino acid sequence alignment showing conservation of the A193 residue across vertebrate lineages. (**C**) Targeting of the A193P substitution in *Tmem98*: a single stranded CRISPR guide oligo was used as a donor template to insert a G > C substitution to create the A193P missense variant, in addition to introducing a silent G > A substitution (and creating a *SapI* restriction site) in codon 194. Coordinates refer to the mouse GRCm38.p6 reference genome. (**D**) Example of genotyping assay for the A193P mutation: a 312 bp PCR amplicon is amplified, spanning codon 193, and digested with the *SapI* restriction enzyme. Alleles carrying the A193P variant also carry a silent G > A variant in *cis*, and are susceptible to cleavage by *SapI*, generating 154 and 158 bp fragments (not resolved on a 1.5% agarose gel).

To validate the functional impact of the human p.(Ala193Pro) variant of *TMEM98*, we used germline CRISPR/Cas9 editing to recreate the equivalent variant in C57BL/6J mice (Fig. 1). Genotyping of F1 progeny (n = 66) weaned from two *Tmem98*^*A193P/wt*^ x *Tmem98*^*A193P/wt*^ breeding pairs demonstrated a wild-type:heterozygote:homozygote ratio of 13:46:7, consistent with no intrauterine lethality (p = 0.06; chi-square test).

We next compared the phenotype of wild-type (*Tmem98*^*wt/wt*^), heterozygous mutant (*Tmem98*^*A193P/wt*^), and homozygous mutant (*Tmem98*^*A193P/A193P*^) mice against the clinical phenotype of a previously reported nanophthalmos case who was heterozygous for the equivalent variant (*TMEM98*^*A193P/*+^)^7^.

**Figure 2:**
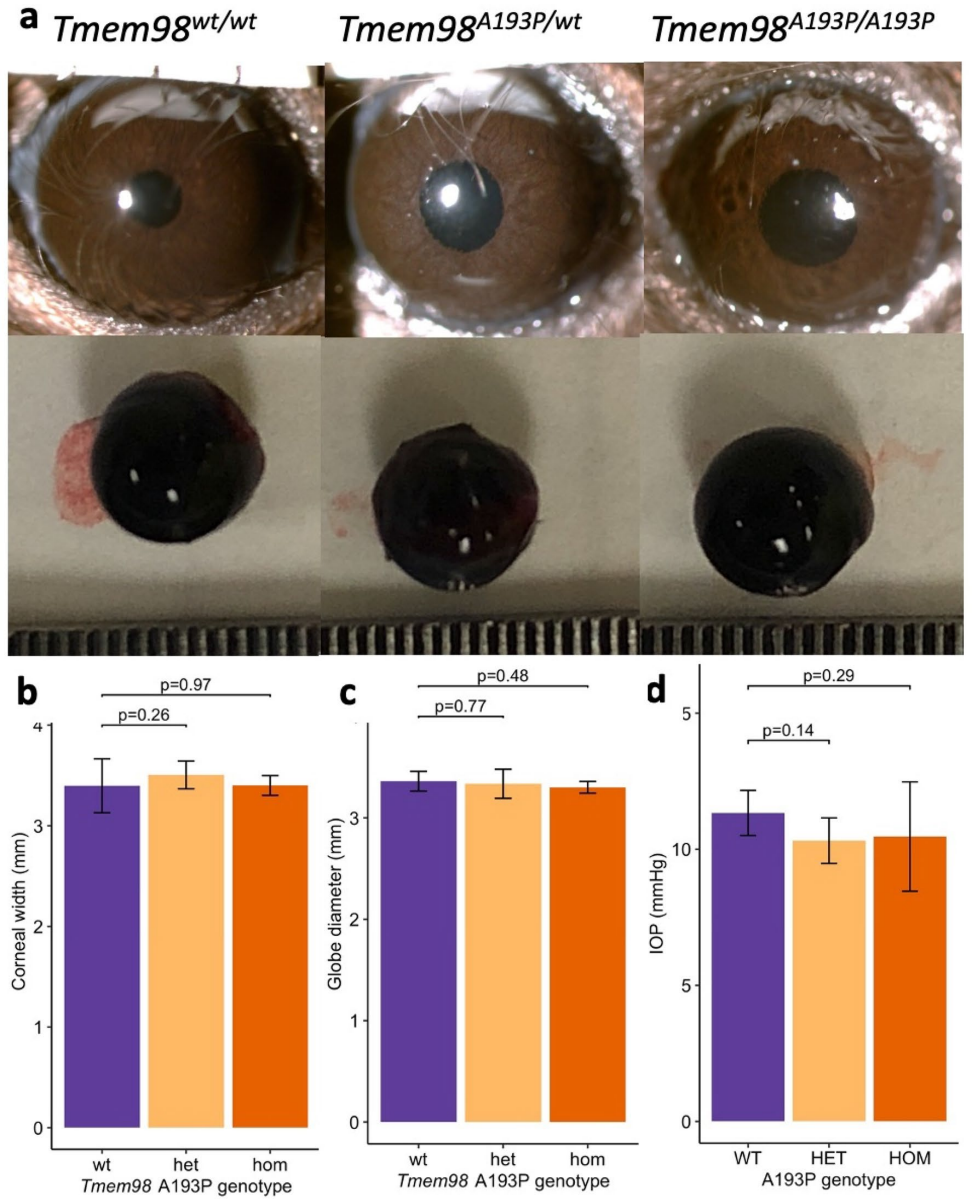
Globe characteristics. (**a**) Colour photographs of the anterior segment (top row) and enucleated globe (bottom row) of each genotype. Each graticule line (bottom row) represents 1/64 of an inch. (**b**) Manual calliper measurements of the corneal diameter. *Tmem98*^*wt/wt*^ (n = 4 eyes), *Tmem98*^*A193P/wt*^ (n = 6 eyes), *Tmem98*^*A193P/A193P*^ (n = 8 eyes). (**c**) Equatorial globe diameter measurements on enucleated globes. *Tmem98*^*wt/wt*^ (n = 4 eyes), *Tmem98*^*A193P/wt*^ (n = 6 eyes), *Tmem98*^*A193P/A193P*^ (n = 8 eyes). (**d**) Intraocular pressure measurements on non-anaesthetised mice. *Tmem98*^*wt/wt*^ (n = 3 eyes), *Tmem98*^*A193P/wt*^ (n = 9 eyes), *Tmem98*^*A193P/A193P*^ (n = 3 eyes). Asterisk: all values represent mean ± 95% CI; compared by hierarchical linear model.

Consistent with other reports, our human *TMEM98*^*A193P/*+^ nanophthalmos case had very short axial lengths in both eyes (RE: 17.1 mm, LE: 17.14 mm), with corresponding high hyperopia (spherical equivalent RE: + 15.5 D, LE: + 15.0 D). However, globe size did not appear to be different in mutant mice, and there were no discernible differences between genotypes in the anterior segment appearance or globe contours, as assessed by two blinded examiners (Fig. 2a). Globe diameters in 7-month-old *Tmem98*^*wt/wt*^ mice (3.4 mm ± 0.08; n = 4 eyes) did not differ from *Tmem98*^*A193P/A193P*^ homozygotes (3.4 mm ± 0.04, p = 0.48; n = 8 eyes) nor *Tmem98*^*A193P/wt*^ heterozygotes (3.5 mm ± 0.05, p = 0.77; n = 6 eyes) (Fig. 2b). Furthermore, corneal diameters of *Tmem98*^*wt/wt*^ controls (3.36 mm ± 0.03; n = 4 eyes) did not differ from *Tmem98*^*A193P/A193P*^ homozygotes (3.3 mm ± 0.04, p = 0.97; n = 8 eyes) nor *Tmem98*^*A193P/wt*^ heterozygotes (3.34 mm ± 0.06, p = 0.26, n = 6 eyes) (Fig. 2c). No eyes were excluded from analysis.

In our human *TMEM98*^*A193P/*+^ nanophthalmos case, the maximum recorded intraocular pressure (IOP) (RE: 33 mmHg, LE: 24 mmHg) was elevated, with gonioscopic examination revealing angle closure, consistent with other reported in patients with *TMEM98* variants^7,9^. The IOP in *Tmem98*^*A193P/A193P*^ homozygous mice (10.5 mmHg ± 0.5) was not significantly different to *Tmem98*^*wt/wt*^ controls (11.3 mmHg ± 0.2) or *Tmem98*^*A193P/wt*^ heterozygotes (10.3 mmHg ± 0.4) (Fig. 2d). Subjective grading of anterior chamber depth using slit lamp examination also did not show a difference between genotypes.

**Figure 3:**
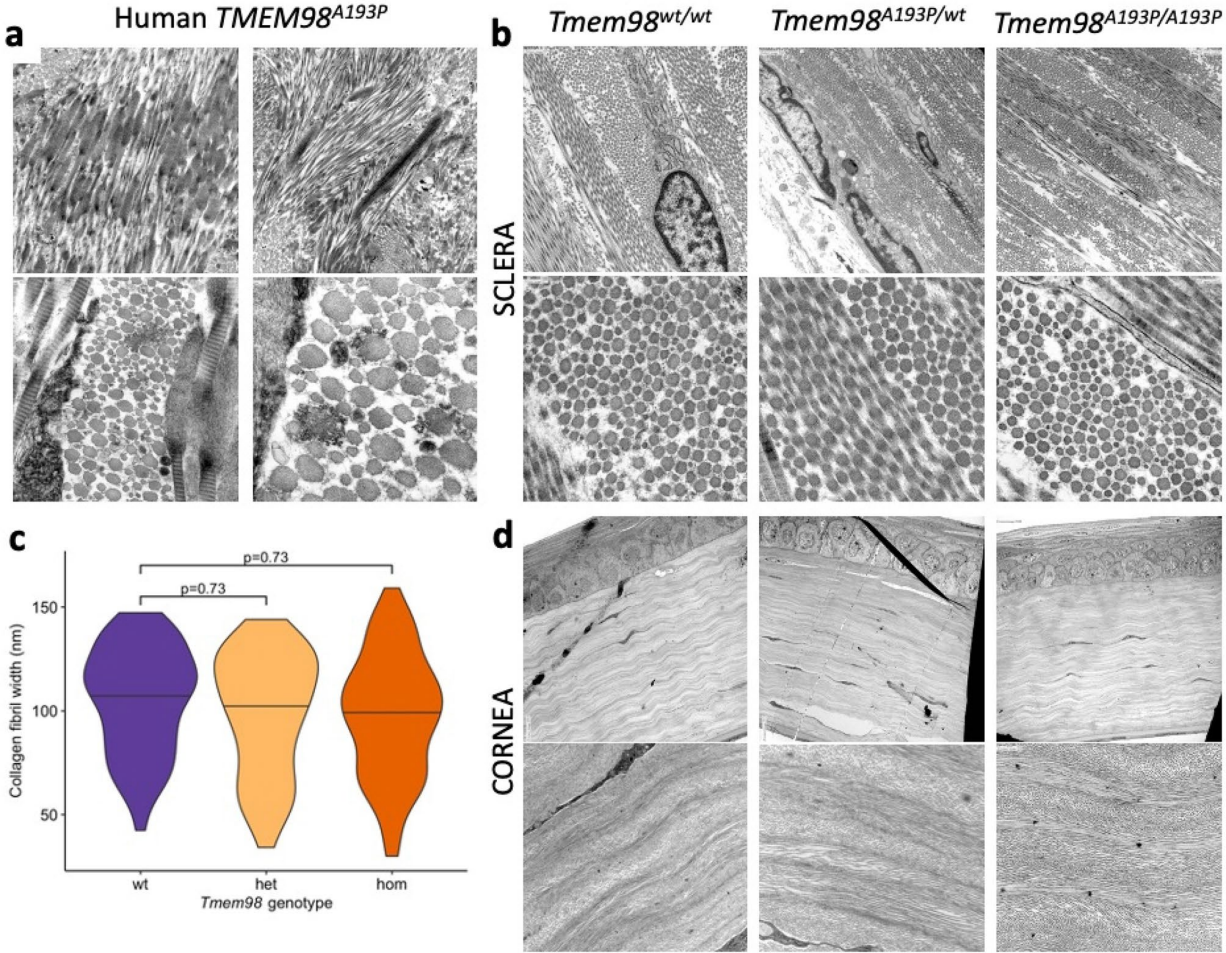
Scleral and corneal collagen fibril phenotypes. (**a**) Electron micrographic (EM) images of the sclera of the human *TMEM98*^*A193P/*+^ case, showing slightly disordered collagen fibres which are arranged in a focally storiform pattern, with some variability in density. Individual collagen fibers display wide variation in the thickness when viewed in cross-section ×15,000 (top panels), ×25,000 (bottom left) and ×80,000 (bottom right) magnification. (**b**) Comparatively, EM images of sclera from each of the three mouse genotypes show no disorganisation and no discernible difference in fibril size. Magnification at ×12,000 (top panels) and ×80,000 (bottom panels). (**c**) Mean and distribution of collagen fibril thickness of each genotype (*Tmem98*^*wt/wt*^, *Tmem98*^*A193P/wt*^, *Tmem98*^*A193P/A193P*^) measured manually on EM images at ×80,000 magnification. All values are presented as means with n = 3 eyes per genotype; and compared by ANOVA. (**d**) EM images of the cornea of each of the three mouse genotypes show no difference in corneal collagen fibril arrangement or lamellae orientation. Magnification at ×1200 (top panels) and ×4000 (bottom panels).

Electron micrographic imaging of sclera (obtained post-mortem) in the human *TMEM98*^*A193P/*+^ nanophthalmos case (Fig. 3a) revealed disorganised collagen bands. Individual collagen fibers displayed wide variation in thickness when viewed in cross-section, and longitudinal sections occasionally displaying thickened bulbous diffuse formations with loss of banding where fibre integrity was disrupted. However, in the *Tmem98* mouse line, all genotypes showed well-organised collagen fibrils with retained fibre integrity and banding (Fig. 3b). In *Tmem98*^*A193P/A193P*^ mice the mean collagen fibril diameter (97.9 nm ± 2.5; n = 3 eyes) was not significantly different to *Tmem98*^*A193P/wt*^ heterozygotes (98.5 nm ± 4.0, P = 0.73; n = 3 eyes) nor *Tmem98*^*wt/wt*^ controls (105 nm ± 2.7, P = 0.73; n = 3 eyes) (Fig. 3c). The corneal collagen fibres in mice also showed no disorganisation or difference between genotypes (Fig. 3d). No eyes were excluded from analysis. These findings suggest that disorganised collagen in the human nanophthalmos case may arise due to abnormally shortened axial length, rather than a primary phenotype due to *TMEM98*-mediated interactions with collagen.

Finally, we compared the appearance of the retina in both human and mouse, using a combination of colour photography, histology, and/or ocular coherence tomography (OCT).

**Figure 4:**
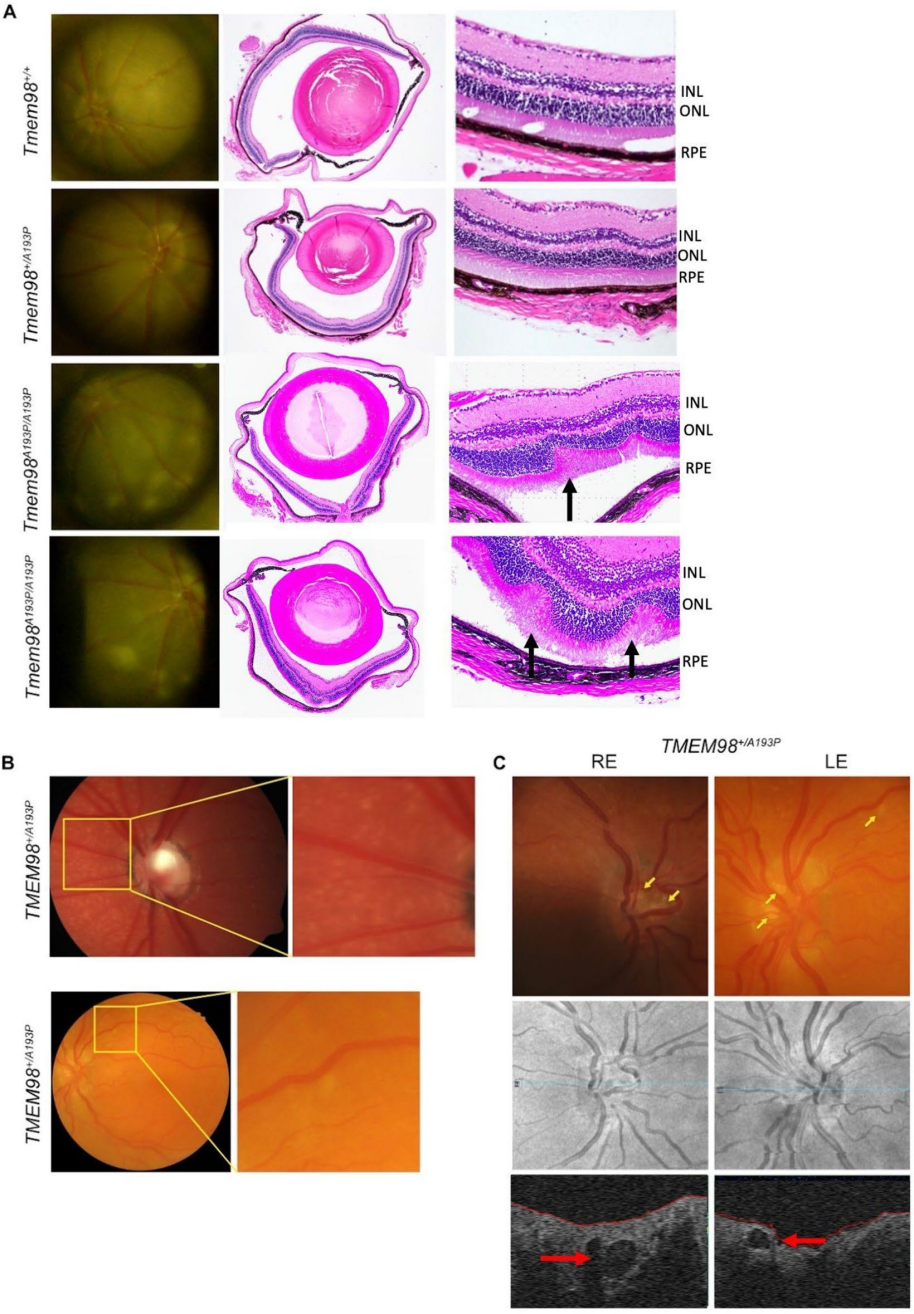
*TMEM98*-associated retinal changes in mice and humans. (**A**) Retinal white dots evident on fundus photography in *Tmem98*^*A193P/A193P*^ homozygous mutant mice. Sections show the histomorphological correlate of a retinal fold (black arrows), characterised by folding of the retina, with only minor associated variation in thickness of the inner nuclear layer (INL). Nerve layer layer thickness is preserved. Bleb formation may be seen as part of retinal fold development. All animals were examined at 7 months of age. (*RPE* retinal pigment epithelium, *ONL* outer nuclear layer). (**B**) Retinal white dots evident on fundus photographs of two human *TMEM98*^*A193P/*+^ nanophthalmos cases, most apparent nasal to the optic disc (top) or at the superotemporal arcade (bottom). (**C**) Bilateral superficial optic disc drusen (yellow arrows) in a human *TMEM98*^*A193P/*+^ case. Top, colour fundus photographs; bottom, cross-sectional OCT image showing disc drusen (red arrows); middle, OCT en face scout image for orientation.

Colour photography of the retinal fundus in 7 month-old mice revealed prominent white dots in *Tmem98*^*A193P/A193P*^ homozyotes, which were not apparent in *Tmem98*^*A193P/wt*^ heterozygotes nor *Tmem98*^*wt/wt*^ controls (Fig. 4A). These were also associated with outer retinal folds on histology, apparently at the level of the outer nuclear layer (Fig. 4A). We also observed retinal white dots in two human *TMEM98*^*A193P/*+^ cases: and in neither case were these white dots correlated with specific features on OCT (Fig. 4B). In at least one *TMEM98*^*A193P/*+^ case, we also observed bilateral superficial optic disc drusen, with a characteristic hyporeflective core on OCT (Fig. 4C)^14^.

## Discussion

Here we perform a cross-species phenotypic comparison in human and mice harboring the same nanophthalmos-associated variant in *TMEM98* (p.(Ala193Pro)). Despite many phenotypic differences, it is clear that TMEM98 is critical for ocular development in both species. In humans, the p.(Ala193Pro) variant can clearly act in a dominant manner, and indeed all *TMEM98*-associated nanophthalmos pedigrees reported thus far show autosomal dominant inheritance. In the mouse however, ophthalmic phenotypes were only evident in *Tmem98*^*A193P/A193P*^ homozygotes.

These findings are consistent with an independent report describing an allelic series of mouse *Tmem98* variants, including the chemically-induced p.(Ile135Thr) allele, the engineered human nanophthalmos variants p.(Ala193Pro) and p.(His196Pro), and the *tm1a* null allele^15^. A subsequent report revealed that although homozygosity for a null allele of *Tmem98* was perinatal lethal in utero, conditional deletion of *Tmem98* in retinal pigment epithelium led to the development of larger eyes^16^.

While homozygosity for a *Tmem98* null allele can lead to development of a larger eye in mouse^16^, compound heterozygosity for a null allele and a nanophthalmos-associated missense variant does not^15^, suggesting that the nanophthalmos-associated variants are unlikely to be null. Similarly, *TMEM98* variants have been positively associated with myopia in genome-wide association studies^12,13^, suggesting that these variants are likely associated with a larger eye, and therefore more likely to resemble the phenotype of null variants in mouse^16^.

Although the p.(Ala193Pro) variant in humans was associated with shorter axial lengths, angle closure, ocular hypertension, and disordered scleral collagen, none of these phenotypes were evident in mice heterozygous or homozygous for the same variant. However, the presence of retinal white dots did appear to be shared across species, corresponding histologically to folds in the outer retinal layers in mice, but with no apparent OCT correlates in humans. The clinical significance of these changes is unclear, although they do suggest that retinal phenotypes in human nanophthalmos and posterior microphthalmos may not exclusively be a consequence of a smaller eye, as has been widely suggested, but that *TMEM98* may itself play a primary role in retinal integrity.

These findings are consistent with other mouse models of human nanophthalmos, where retinal phenotypes predominate in mouse mutants of *PRSS56*^2^, *MFRP*^4,5^, and *MYRF*^6^, the last of which also has intricate functional links with TMEM98^16,17^. Whether this is a reflection of strain-specific genomic modifiers^18^, or species-differences in ocular development and anatomy, is unknown.

## Materials and methods

For human subjects, patients and family members provided written informed consent. Study protocol were approved by the Southern Adelaide Clinical Human Research Ethics Committee. The study was conducted in accordance with the revised Declaration of Helsinki and followed the National Health and Medical Research Council statement of ethical conduct in research involving humans.

For mouse colonies, CRISPR/Cas9 editing was used to create the *Tmem98* c.577G > C p.(Ala193Pro) variant (ENSMUST00000040865.8). This mouse line is designated as C57BL/6J-*Tmem98*^*em1Siggs*^, and abbreviated as either *Tmem98*^*wt/wt*^, *Tmem98*^*A193P/wt*^ or *Tmem98*^*A193P/A193P*^ mice for wt, heterozygotes and homozygotes respectively. The online tool (http://benchling.com) was used to search for appropriate CRISPR guide sequences. The most appropriate guide was determined by total score, cut site within the target gene, and number of off-target mismatches. The CRISPR guide sequence CCAATCACTGTCTGCCGCTG (forward strand) was determined to be most suitable. A single stranded oligo was used as a donor template to insert the G > C point mutation converting codon 193 from alanine to proline, as well as the silent mutation G > A in codon 194 (to introduce a *SapI* restriction site 6 bp from the p.(A193P) mutation). The donor template contained 60 bp homology arms either side of the mutations. Cas9 protein was microinjected into C57BL/6J mouse zygotes together with guide RNA and donor oligo, and implanted into pseudopregnant females. Genotyping was initially performed by capillary sequencing of a PCR amplicon (Fwd:CAAAGTTCCCCACTTTCTACAG, Rev:AGGAAGTAGAAGGCTCGCCC). Once germline transmission of the correct variant was confirmed, further genotyping was performed by *SapI* restriction digest of the same PCR amplicon (wild-type allele 312 bp, mutant allele 154 + 158 bp). Animal ethics approval was obtained from the Flinders University Institutional Biosafety Committee (2015–18) and Animal Welfare Committee (904/16), in accordance with the South Australian Animal Welfare Act (1985) and the Australian Code for the Care and Use of Animals for Scientific Purposes (2013, 8th Edition). Animal use and outcomes were reported in accordance with the ARRIVE guidelines.

Mouse phenotyping included intraocular pressure, measured on non-anaesthetised mice with an Icare TONOLAB rebound tonometer (Icare, Finland), taking the median of three consecutive pressure measurements across a single eye. Prior to retinal imaging, mice were anesthetised with intraperitoneal ketamine (50 mg/kg) and xylazine (5 mg/kg), and eyes dilated with 1% tropicamide and 2.5% phenylephrine drops. Retinal fundus photography was performed after application of lubricant to each eye, overlaid with a glass cover slide. The coverslip neutralised the dioptric power of the cornea-air interface and allowed clear fundus photographs of the retinal dots. Measurements of corneal diameter and globe diameter were performed by a blinded assessor using digital callipers (ImageJ; NIH) on standardised photographs of the anterior segment or enucleated globe, each containing a reference graticule.

Histology was performed on freshly enucleated eyes that were fixed in 4% paraformaldehyde at room temperature for at least 24 h, dehydrated, embedded in paraffin, cut into 5 μm sections, and stained with hematoxylin and eosin.

Electron microscopy was performed by first fixing enucleated eyes in 2% glutaraldehyde in 0.1 M sodium cacodylate buffer (pH 7.4). Samples were processed commercially (Electron microscopy division, SA Pathology, South Australia) using a scanning electron microscope. The diameter of collagen fibrils was measured by a blinded assessor using digital callipers (ImageJ; NIH) on 1000 × 1000 pixel regions of interest randomly sampled from 80,000× magnification EM images of cross-sectional collagen fibres. Collagen fibril organisation was subjectively assessed against the human nanophthalmos case EM images by an experienced ocular histopathologist (SK).

All data preparation and analyses were performed using R (version 3.5.1, RCore Team, Austria). Measurements of globe diameter, corneal diameter and collagen fibril diameter were compared between genotypes using a linear model with mixed effects to account for paired eye data. The significance level (alpha) was set at 0.05 for two-tailed hypothesis testing.

## Data Availability

All data generated or analysed during this study are included in this published article.
